# Establishment of a Comprehensive Cardiac Amyloidosis Center in a Community Hospital Setting

**DOI:** 10.31083/j.rcm2502061

**Published:** 2024-02-05

**Authors:** Prabin Phuyal, Sabahat Bokhari

**Affiliations:** ^1^Department of Medicine, Saint Peter’s University Hospital/Rutgers Robert Wood Johnson Medical School, New Brunswick, NJ 08901, USA; ^2^Division of Cardiology, Department of Medicine, Robert Wood Johnson University Hospital, New Brunswick, NJ 08901, USA

**Keywords:** amyloidosis, cardiac amyloidosis, screening, transthyretin

## Abstract

Cardiac amyloidosis is a great masquerader that often results in misdiagnosis of this condition. Early clinical recognition is crucial for timely therapeutic interventions to improve survival in patients with cardiac amyloidosis. Currently, Food and Drug Administration (FDA)-approved medications work best if started early in the disease. Thus, to increase identification, disease awareness, expertise in diagnostic techniques, and a multidisciplinary team approach is essential. The majority of the patients (~90%) in the United States are treated in community hospitals, thus, it would be helpful for these hospitals to have their own designated, comprehensive cardiac amyloidosis center to provide care to the patients who are widespread in the community. Most of these patients are elderly, and it is difficult for these patients to travel long distances to academic amyloid centers. Our manuscript aims to provide a path to the development of cardiac amyloid centers at community hospitals.

## 1. Overview of Cardiac Amyloidosis 

Cardiac amyloidosis (CA) is an infiltrative cardiomyopathy that commonly causes heart failure and results from the extracellular deposition of misfolded proteins, which configures the insoluble beta-pleated sheet structures called amyloid fibrils. Over 30 amyloid precursor proteins are identified, nine of which frequently cause CA, of which over 95% result from the amyloid light chain (AL) and amyloid transthyretin (ATTR) [1, 2, 3]. AL arises from misfolded monoclonal immunoglobulin light chains (produced from clonal plasma cells), and ATTR arises from misfolded transthyretin (TTR) protein (produced predominantly in the liver), resulting in AL-CA and ATTR-CA respectively. ATTR can occur either through mutation in transthyretin (variant-ATTR/ATTRv) or age-related processes without mutation in transthyretin (wild-type ATTR/ATTRwt). Thus, AL-CA, ATTRwt-CA, or ATTRv-CA are the most common types of CA [4]. Valine-to-isoleucine substitution at position 122 (Val122Ile) is the most common mutation causing ATTRv-CA in the USA, seen in 3.5% of African Americans, especially of Western African origin [5]. AL-CA is rare, with an annual incidence estimated to be 1 in 75,000 to 100,000 as of 2015, with cardiac involvement present in about 75% of patients [4]. The true prevalence of ATTR-CA in the population is unknown. However, several autopsy studies have shown the prevalence of ATTRwt amyloid deposits up to 20–25% in octogenarians and 37% in people over age 95 [2]. ﻿Cardiac involvement determines the prognosis in patients with amyloidosis, with median survival rates ranging from 3.6 to 4.8 years for ATTRwt-CA, 2.6 years for ATTRv-CA due to Val122Ile, and 5.8 years for ATTRv-CA due to other mutations and less than six months for AL-CA, if left untreated [4, 6]. Therefore, early clinical recognition is crucial for timely therapeutic interventions to improve survival in patients with CA.

## 2. Need for Cardiac Amyloidosis Centers

CA is a great masquerader because of its marked heterogeneity in terms of cardiac phenotype or systemic involvement, often resulting in misdiagnosis for other more common diseases such as hypertensive heart disease, aortic stenosis, or hypertrophic cardiomyopathy [7, 8]. As a result, many patients experience significant delays in receiving a correct diagnosis, with 31.8% of patients reporting consulting more than five physicians before receiving a correct diagnosis of amyloidosis [9]. To overcome these challenges, a multidisciplinary team approach involving expertise such as cardiologists, hematologists, neurologists, nephrologists, nuclear imaging experts, pathologists, genetic counselors, nurses, and specialty pharmacists is necessary. This approach involves identifying diagnostic clues or red flags for CA, screening high-risk patients with appropriate diagnostic tools and their appropriate interpretation, offering genetic testing and counseling, and providing treatment, including advanced therapies such as cardiac transplantation, thus highlighting the need for CA centers to provide all the facilities in one integrated setting.

The present era of CA is advancing significantly, allowing patients to survive for over a decade with treatment [4, 10]. Thus, patients with CA will require ongoing care from specialists to manage their chronic condition. However, not all patients have access to multidisciplinary care because of the limited availability of CA centers. For instance, as per the American Hospital Association’s annual survey for 2021, there are 6129 hospitals in the United States, of which 5157 are community hospitals [11]. As of April 2023, only 47 centers in the United States participated in the Transthyretin Amyloidosis Outcomes Survey (THAOS) registry, the largest global, multicenter, longitudinal observational survey [12]. This poses a challenge for patients who are spread out across this country to travel to these centers to seek medical care, as travel, in particular, is reported as the most significant challenge by nearly one-third of patients [13]. To make this care more accessible, more CA centers should be established in the community near where patients live. This will help reduce travel time, increase awareness among community physicians, prompt more referrals, and ultimately result in more CA screening and identification. To provide care to the growing patient population, there will also be a need for more specialists in CA, which CA centers can help provide by establishing fellowship programs allowing opportunities for physicians to train in CA. Moreover, as of 2022, only one FDA-approved disease-directed therapy, Tafamidis, is available for ATTR-CA; thus, more CA centers are needed to provide a platform for ongoing and future clinical trials in this field [4, 14].

Our objective is to provide a structured approach and steps to help establish a new comprehensive CA center in a community-based hospital setting.

## 3. Estimate Institutional Needs and Benefits

The first step in establishing a CA center is demonstrating its necessity and benefits to the hospital. One reliable way to do this is by estimating the number of undiagnosed CA among patients already registered within the hospital system. For instance, the Medicare population has an estimated 17% prevalence of heart failure, with approximately 50% of those cases being heart failure with preserved ejection fraction (HFpEF) [15, 16]. Assuming there are 100,000 Medicare patients enrolled in the hospital system, an estimated 17,000 patients may have heart failure, with 8500 patients potentially having HFpEF. Epidemiological studies suggest a 12–13% prevalence of CA in HFpEF, which means around 1100 patients may have undiagnosed CA [17, 18]. An estimate of undiagnosed cases of CA can then be made by checking how many of these patients are receiving treatment for amyloidosis. As CA worsens heart failure and a result of an observational study showed that in Medicare beneficiaries, the per-patient cost from single heart failure hospitalization averaged around $14,600 with a mean length of stay for heart failure-related hospitalization being 7.07 days, this estimate can help assess the economic burden to the hospital from potential undiagnosed CA [19]. Thus, screening for CA and treating the affected ultimately results in significant financial improvement for the hospital by preventing hospitalizations. This estimation is critical to garner attention and get continuous support from the hospital administration.

After estimating the potential patients with undiagnosed underlying CA who are already in the hospital system, creating variables in the electronic medical records will be an important and effective way to identify these patients.

## 4. Availability of Multidisciplinary Expertise 

After identifying the institutional need, recruiting a dedicated group of professionals with specific expertise is essential. This team should include specialists such as cardiologists, hematologists, neurologists, nephrologists, nuclear imaging experts, pathologists, genetic counselors, nurses, and specialty pharmacists. A patient navigator is also crucial in assisting patients with navigating complex healthcare systems, overcoming access barriers, and effectively managing medical paperwork, ensuring timely initiation and continued treatment adherence. In addition to the expertise, it is also equally essential to establish the necessary infrastructure.

## 5. Improve Awareness 

Improving awareness among healthcare professionals is crucial in the early detection and intervention of CA [20, 21, 22]. Primary care physicians and various specialists, including cardiologists, hematologists, neurologists, gastroenterologists, nephrologists, and orthopedics, are among those who should be made aware of CA. They should be educated about the diagnostic clues or red flags that help identify high-risk patients under their care. Physicians should also be aware of the appropriate assessments and diagnostic tools. The institutional marketing department should inform the community physicians about the CA center. By doing so, patients at high risk can be referred early to cardiac amyloid specialists for screening of CA and treatment when the disease is established.

To increase awareness among physicians, educational programs like grand rounds, morbidity and mortality conferences, and regional and national meetings can be utilized to educate them on CA and its diagnostic clues or red flags. It is also crucial to raise awareness among the general public, especially African Americans who have a higher risk of developing ATTR-CA. One effective way to do this is by spreading or circulating educational videos at community events or coverage through local media outlets. This approach will promote medical care-seeking behavioral change. It will also encourage the patients to participate in ongoing and future clinical trials while supporting patients and their families.

## 6. Implementation of Diagnostic Tools and Assessment 

### 6.1 Identification of Diagnostic Red Flags 

A high level of suspicion is fundamental in making the diagnosis of CA. Knowing typical patterns of disease presentation, diagnostic clues or red flags, and commonly affected demographics will help to order specific laboratory and imaging studies. For instance, if an older individual is hospitalized for heart failure and has elevated baseline troponin levels or N-terminal pro-B-type natriuretic peptide (NT-proBNP) levels that don’t match the clinical context, it could be a sign of ATTR-CA. Other indicators include hypertension that eventually goes away or needs down-titration of antihypertensives and an inability to tolerate beta-blockers or ACE inhibitors/angiotensin receptor blockers. Early clues or red flags to ATTR-CA may include bilateral carpal tunnel syndrome, lumbar spinal stenosis, previous orthopedic procedures, and spontaneous biceps tendon rupture [23, 24].

### 6.2 Whom to Screen 

**Fig. 1.**
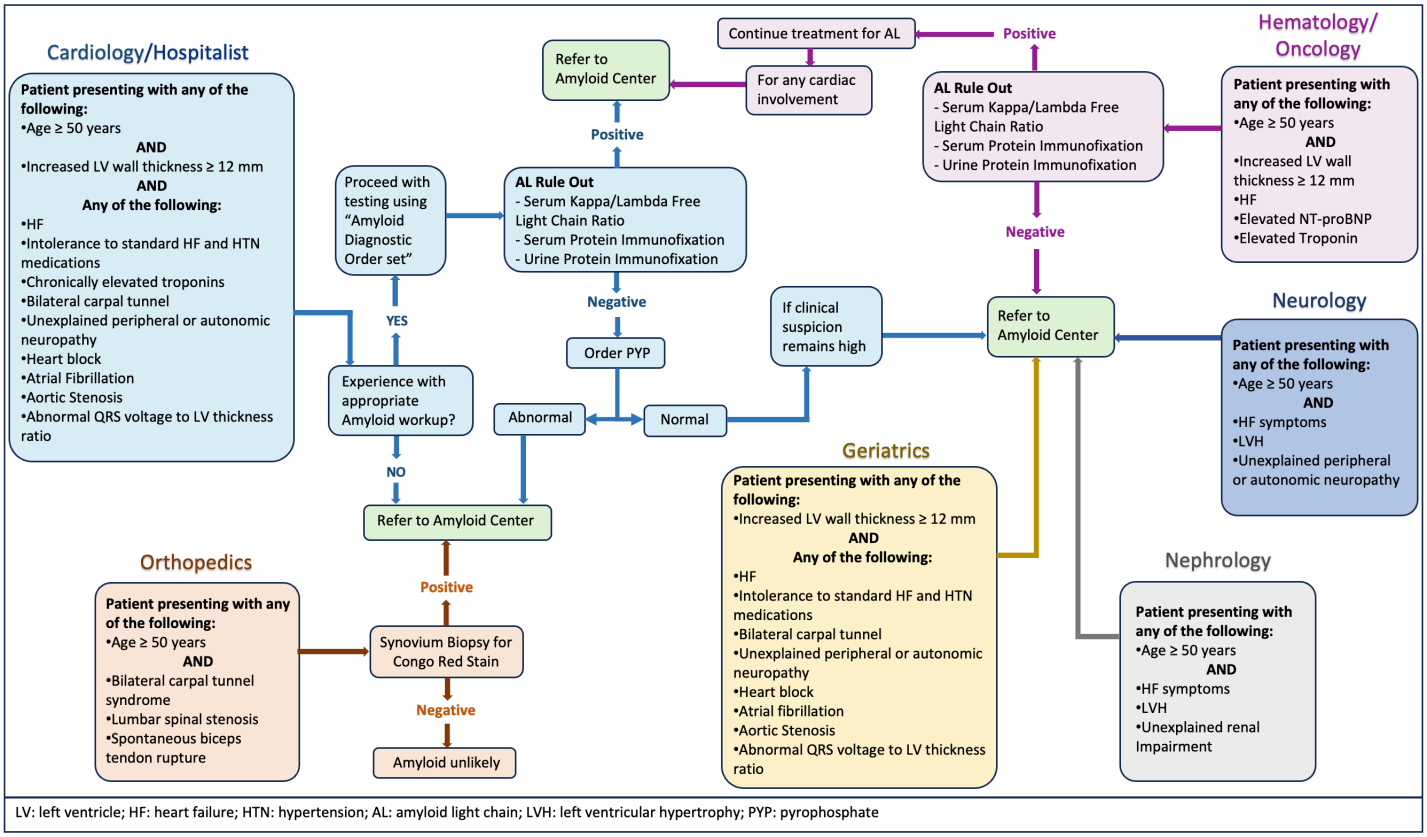
**Multispecialty comprehensive approach and diagnostic red flags or clinical clues for cardiac amyloidosis**.

Screening the right patient population is the bottom line for accurately identifying the disease. It is essential to consider the pretest probability of finding the disease before conducting the screening. For instance, non-invasive methods using cardiac scintigraphy can accurately diagnose ATTR-CA without needing a biopsy in specific clinical scenarios with about 100% specificity [25]. However, failing to consider the pretest probability and conducting the screening incorrectly can decrease the accuracy of diagnostic tests. Additionally, a lack of expertise in interpreting test results can lead to false-positive or negative outcomes, reducing the sensitivity and specificity of the test. Here, we propose a framework for physicians, including clinical, electrocardiographic, echocardiographic, and imaging diagnostic clues or red flags recommended by the experts in the field that should be considered while screening for CA, elaborated in Fig. 1.

### 6.3 How to Screen 

The diagnostic testing process starts with a high index of clinical suspicion based on clinical, electrocardiographic, echocardiographic, and imaging diagnostic clues or red flags to CA. The diagnostic algorithm should start with screening for monoclonal proteins to assess for any underlying plasma cell dyscrasias, as the presence or absence of monoclonal proteins determines the appropriate diagnostic pathway. While cardiac scintigraphy is a useful noninvasive tool in diagnosing ATTR-CA, it is essential to note that 10% of patients with AL-CA may also show consistent cardiac uptake in scintigraphy. Thus, the interpretation of cardiac scintigraphy without checking for monoclonal protein can often lead to misinterpretation and inaccurate diagnosis and is not reliable for distinguishing between ATTR-CA and AL-CA [4, 25].

The initial screening methods for monoclonal proteins include serum-free light chain (sFLC) assay and serum and urine immunofixation electrophoresis (SIFE and UIFE). It is crucial to perform serum and urine electrophoresis with immunofixation to detect low levels of circulating monoclonal proteins. If no monoclonal proteins are detected in SIFE or UIFE, and the sFLC ratio is within the normal range, in that case, AL-CA can be reliably ruled out with a ~99% negative predictive value and the specificity of cardiac scintigraphy for diagnosis of ATTR-CA is about 100% [4, 6, 25]. However, when monoclonal proteins are detected or the sFLC ratio is abnormal, then in such circumstances, a hematologist consultation is needed for the correct interpretation of the results, and a biopsy of the affected organ becomes absolute. AL-CA can be diagnosed only by demonstrating the deposits of AL amyloid fibrils in the affected organs.

Imaging is the next crucial step in the algorithm after obtaining laboratory results. Echocardiography and cardiac MRI can provide helpful information, but neither can diagnose CA or distinguish between AL-CA and ATTR-CA. Cardiac scintigraphy with technetium (Tc)-labeled bisphosphonates is the most useful option to confirm the presence of ATTR cardiac amyloid deposits after ruling out AL amyloidosis. Among the available radiotracer options for scintigraphy including 99mTc-labeled hydroxy methylene diphosphonate (HMDP), 99mTc-labeled diphosphono-1,2-propanodicarboxylic acid (DPD), and 99mTc-labeled pyrophosphate (PYP), Tc-PYP is the most commonly used radiotracer in the United States. The Tc-PYP scan is taken with both planer images, followed by a single-photon emission computed tomography (SPECT) study, which confirms the myocardial uptake of radiotracers [26]. An endomyocardial biopsy is required in scenarios where a monoclonal protein is detected, cardiac imaging is unavailable, or there is strong suspicion for CA despite negative or unclear results of cardiac scintigraphy [4, 6].

### 6.4 Role of Genetics 

When diagnosis of ATTR-CA is confirmed with Tc-PYP cardiac scintigraphy, the next crucial step is to offer genetic counseling to patients and arrange for TTR genetic testing or sequencing [2]. This is crucial in distinguishing between ATTRwt-CA and ATTRv-CA, as clinical profiles are insufficient for differentiation. If the variant identified in the TTR gene sequencing is pathogenic or likely pathogenic, a three-generation (or more) family history should be obtained, and the at-risk first-degree healthy relatives should undergo cascade testing [4]. These healthy relatives should be offered pre- and post-genetic counseling sessions, including the implications of genetic testing on insurance and employment and therapeutic options based on testing results.

### 6.5 Multidisciplinary Collaboration

Multidisciplinary collaboration is essential for providing an opportunity for all specialists to collaborate and coordinate in providing expert-level comprehensive care to patients with CA in a single integrated setting. Cardiologists are the primary care providers for patients with ATTR-CA. At the same time, hematologists are often the primary care providers for patients with AL-amyloidosis in collaboration with cardiologists for cardiac involvement. The primary care physician must recognize the signs and symptoms of ATTR-CA and refer patients to a cardiologist for further evaluation. Cardiologists must possess knowledge of clinical presentation and diagnostic clues or red flags of CA and be able to integrate the biopsy and Tc-PYP imaging into the diagnostic algorithm for patients with suspected ATTR-CA after ruling out AL-CA. Nuclear cardiologists must understand and adhere to proper Tc-PYP procedures, standardized testing protocols, and reporting in the hospital. Genetic counselors must provide counseling to the patients and their families and understand genetic testing and screening. Collaboration with gastroenterologists and neurologists is necessary to manage complications from gastrointestinal amyloid deposition, autonomic dysfunction, and polyneuropathy, respectively. Many patients with lumbar spinal stenosis and carpal tunnel syndrome can also develop CA, so orthopedics should also be able to identify patients who are at risk. Nephrologists must be involved as kidney involvement is present in about 70% of patients with amyloidosis, particularly in AL [4, 27]. Multidisciplinary collaboration is also crucial in determining if any systemic involvement contraindicates a heart transplant in cases of advanced heart failure.

## 7. How to Sustain the Center 

**Fig. 2.**
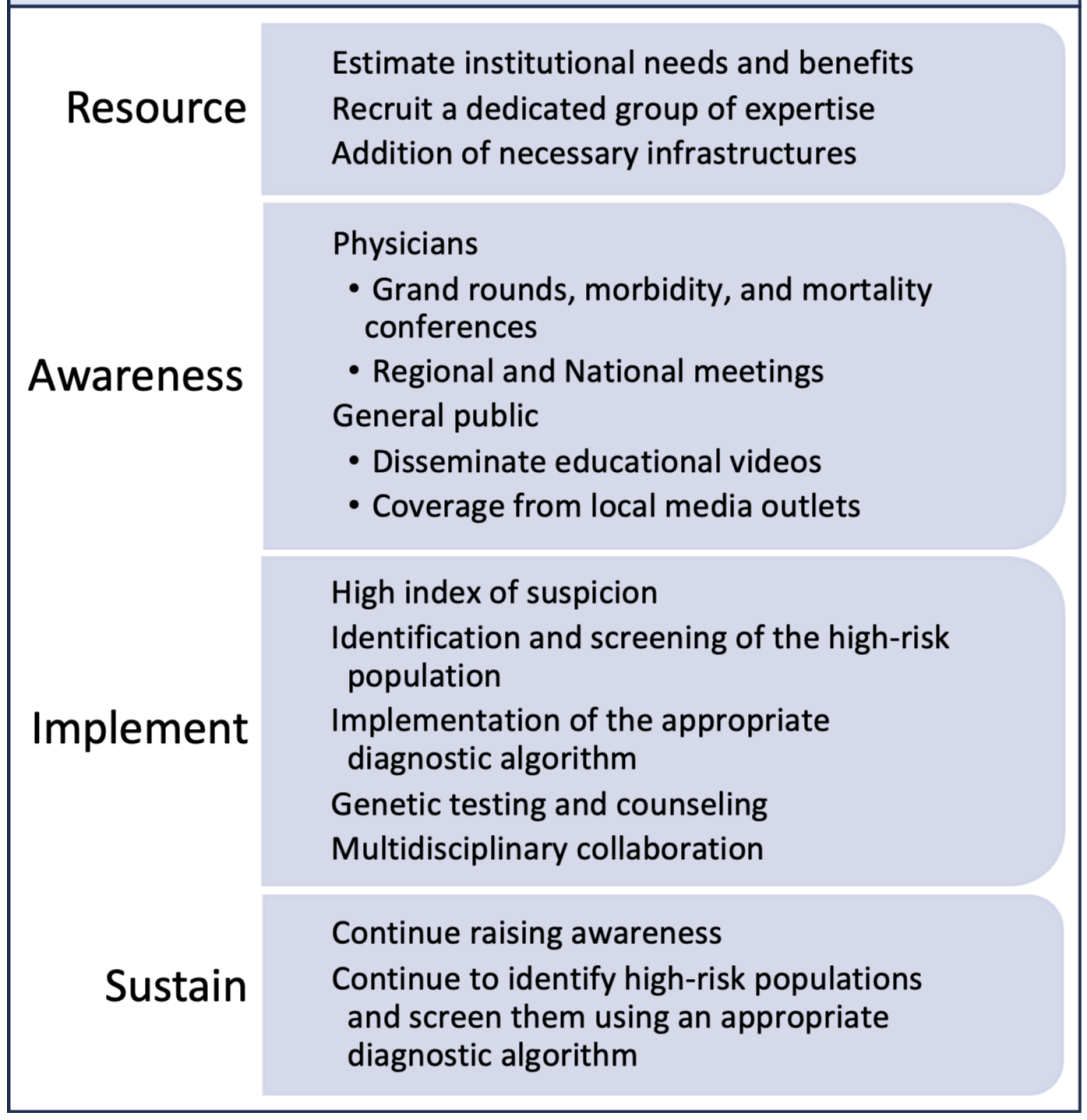
**Steps to establish and sustain an amyloidosis center**.

Consistently implementing the above steps is crucial for maintaining the center’s growth after its establishment, elaborated in Fig. 2. Educating community physicians periodically about the disease and its diagnostic clues or red flags will continue to identify high-risk patients and their early referral to the CA center. Educating patients about the condition will increase their likelihood of enrolling in medical care and participating in research. Proper use of the diagnostic algorithm and building an order set in the electronic medical record system to order diagnostic tools easily is imperative. Continuous screening is essential for identifying more patients and thus continues to improve the hospital’s financial burden, allowing for the growth and expansion of the CA center.

## 8. Conclusions

The majority of the patients seek medical care at the community hospital. Thus, it would be helpful to have a CA center in a community hospital setting to provide comprehensive care to the growing population of patients with CA. This manuscript aims to provide community-based hospitals with a framework to help establish their own designated, comprehensive CA center.

## Abbreviations

CA, Cardiac amyloidosis; AL, amyloid light chain; TTR, transthyretin; ATTR, amyloid transthyretin; AL-CA, light chain cardiac amyloidosis; ATTR-CA, transthyretin cardiac amyloidosis; ATTRv-CA, variant transthyretin cardiac amyloidosis; ATTRwt-CA, wild-type transthyretin cardiac amyloidosis; sFLC, serum-free light chain; Tc-PYP, 99mTc-labeled pyrophosphate.
